# 1989年-2008年中国肺癌发病性别、城乡差异及平均年龄趋势分析

**DOI:** 10.3779/j.issn.1009-3419.2013.09.02

**Published:** 2013-09-20

**Authors:** 仁强 韩, 荣寿 郑, 思维 张, 鸣 武, 万青 陈

**Affiliations:** 1 210009 南京，江苏省疾病预防控制中心 Jiangsu Provincial Center for Disease Prevention and Control, Nanjing 210009, China; 2 100021 北京，国家癌症中心 National Cancer Center, Beijing 100021, China

**Keywords:** 肺肿瘤, 发病相对指数, 平均发病年龄, 中国, Lung neoplasms, Incidence rate ratio, Average age, China

## Abstract

**背景与目的:**

近20年来，中国肺癌的发病水平呈明显上升趋势，但其性别和城乡发病差异以及平均发病年龄的变化趋势并不清楚。本研究将对1989年-2008年中国肺癌发病率在性别、城乡差异和平均发病年龄的变化趋势进行分析。

**方法:**

利用全国1989年-2008年肿瘤登记地区的肺癌发病数据和人口数据，按照不同性别、不同地区（城乡）分层，运用*Poisson*回归模型和线性回归模型，分析发病率的性别比值、城乡比值和平均发病年龄的变化特征。

**结果:**

和1989年相比，2008年肺癌发病率男女比值由2.47降低到了2.28，城乡比值由2.07下降到了1.14，男性平均发病年龄由65.32岁升高到了67.87岁，女性平均发病年龄由65.14岁升高到了68.05岁。肺癌发病的男女差异和城乡差别逐渐缩小，平均发病年龄逐年升高，变化趋势均有统计学意义。

**结论:**

近20年，中国肺癌发病的性别和城乡差异明显缩小，发病年龄趋于老龄化，应针对肺癌发病现状开展更有效的防治研究工作。

## Abstract (en)

**Background and objective:**

The lung cancer incidence was significantly increased in the past two decades in China. But the secular trend of lung cancer incidence difference between gender (male and female), area (urban and rural) and average age was not clear. The aim of this study is to analyze the trend of lung cancer incidence rate ratio between gender, area and average age in China during 1989-2008, and provide some inspiration for lung cancer prevention and control activities.

**Methods:**

Cancer registry data and population data in China from 1989 to 2008 were collected by the National Cancer Registration Center. Stratified for gender and area, *Poisson* regression model was used to estimate annual lung cancer incidence rate ratio between male and female, urban and rural areas. The average age of lung cancer incidence was calculated. Linear regression was used to model the trend of annual incidence rate ratio and average age over time.

**Results:**

Incidence rate ratios of lung cancer between male and female, urban and rural areas were significantly decreased from 2.47 and 2.07 to 2.28 and 1.14 during 1989-2008, respectively. Meanwhile, the average age of lung cancer incidence among male and female dramatically increased from 65.32 and 65.14 to 67.87 and 68.05, respectively.

**Conclusion:**

The difference of lung cancer incidence between male and female, urban and rural areas remarkably decreased from 1989 to 2008. The average age of lung cancer occurrence gradually increased. Researches, prevention and control activities on lung cancer with consideration of new incidence pattern should be strengthened.

肺癌是世界上最常见恶性肿瘤之一。据世界卫生组织估计，2008年全球约新发肺癌161万例，死亡138万例，分别占当年全部恶性肿瘤发病和死亡的12.7%和18.2%，肺癌新发病和死亡高居各种恶性肿瘤之首^[1]^。近20年来，随着我国社会经济的飞速发展，城市化、工业化进程的不断加快，我国居民的生活方式和生活环境均发生着急剧的变化，以往在发达国家高发的肺癌在我国的发病率也呈明显上升趋势^[2]^。由于我国城乡经济发展、生活方式转变和生存环境改变的程度不同，以及医疗条件和健康相关知识知晓水平的不同，肺癌的发病也存在明显城乡差异^[3]^，但城乡差异随时间的变化尚无报道。而由于肺癌危险因素，如吸烟、被动吸烟、职业危险因素等在不同性别间暴露水平的不同，以及生理结构的差异，男性和女性的肺癌发病水平存在差异，但不同性别间发病差异的长期变化趋势尚无研究。另外，以往研究^[4]^表明，人口老龄化是导致我国肺癌发病率不断升高的主要原因，但随着人口老龄化进程的加快和肺癌发病水平的升高，我国肺癌平均发病年龄的变化趋势尚未知晓。因此，本研究利用1989年-2008年我国肿瘤登记发病数据，分析20年间不同地区、不同性别肺癌发病差异的变化以及肺癌平均发病年龄的变化，为掌握我国肺癌的流行趋势，确定肺癌防治重点人群，以及制定切实可行的肺癌防治策略提供理论基础。

## 1 资料与方法

### 1.1 资料来源

从全国肿瘤登记数据库中提取1989年-2008年各登记点历年的肺癌发病数据，进行合并和整理。20年间，肿瘤登记处由1989年的10个增加到2008年的41个，累计覆盖人口711, 843, 051人年。

### 1.2 统计方法

按照不同地区（城市/农村），性别（男性/女性），年龄组（0岁，1岁-4岁，5岁-9岁，10岁-14岁，15岁-19岁，20岁-24岁，25岁-29岁，30岁-34岁，35岁-39岁，40岁-44岁，45岁-49岁，50岁-54岁，55岁-59岁，60岁-64岁，65岁-69岁，70岁-74岁，75岁-79岁，80岁-84岁和85岁以上共19个年龄组）和年份（1989年-2008年）进行分层。应用SAS统计软件Genmod模块拟合*Poisson*回归模型，计算每年发病率的城乡比值和性别比值（incidence rate ratio, IRR）以及95%的置信区间（分别调整年龄、地区和性别），并通过线性回归模型对比值的变化趋势进行统计学检验。平均发病年龄按照年龄组的中位值和年龄组病例数进行计算，85岁组按87.5岁计算，并检验20年间平均发病年龄的变化趋势。

## 2 结果

### 2.1 肺癌发病率男女性别比变化

1989年-2008年我国肿瘤登记地区肺癌发病率呈上升趋势，其中男性和女性发病率均有不同程度升高，且女性升幅高于男性；肺癌发病率的男女性别比也由1989年的2.47降低到2008年的2.28，呈明显下降趋势（*P* < 0.001）。20年间我国登记地区的肺癌发病率均为男性高于女性，其中农村地区的性别差异一直大于城市；但农村地区肺癌发病的性别比20年间无明显变化趋势（*P* > 0.05），1989年和2008年均为2.66；而城市地区则呈现明显下降趋势（*P* < 0.001），肺癌发病率的男女性别比由1989年的2.45降低到2008年的2.21（表 1，图 1和图 2）。

**1:** 1989年-2008年中国肺癌发病率男女比值 Incidence rate ratio (IRR) of lung cancer between male and female in China, 1989-2008

Year	All areas			Urban			Rural	
	IRR	95%CI		IRR	95%CI		IRR	95%CI
1989	2.47	2.35-2.59		2.45	2.33-2.57		2.66	2.24-3.15
1990	2.46	2.35-2.57		2.44	2.32-2.56		2.63	2.25-3.08
1991	2.53	2.41-2.65		2.51	2.39-2.63		2.76	2.33-3.26
1992	2.49	2.37-2.60		2.43	2.32-2.55		3.19	2.69-3.78
1993	2.36	2.26-2.47		2.32	2.22-2.43		2.90	2.46-3.43
1994	2.42	2.32-2.53		2.36	2.25-2.47		3.36	2.83-4.00
1995	2.32	2.22-2.42		2.23	2.14-2.34		3.50	2.98-4.11
1996	2.29	2.19-2.39		2.22	2.12-2.32		3.17	2.70-3.72
1997	2.34	2.25-2.45		2.27	2.17-2.37		3.23	2.77-3.76
1998	2.26	2.18-2.34		2.12	2.04-2.21		3.16	2.86-3.48
1999	2.21	2.13-2.29		2.10	2.02-2.18		2.87	2.61-3.15
2000	2.24	2.17-2.32		2.18	2.10-2.26		2.65	2.42-2.90
2001	2.30	2.23-2.38		2.21	2.14-2.29		2.89	2.64-3.15
2002	2.29	2.22-2.37		2.20	2.12-2.28		2.94	2.69-3.22
2003	2.15	2.10-2.21		2.09	2.03-2.15		2.56	2.39-2.75
2004	2.21	2.15-2.27		2.14	2.08-2.20		2.58	2.42-2.76
2005	2.25	2.20-2.31		2.15	2.09-2.22		2.79	2.62-2.98
2006	2.27	2.22-2.33		2.21	2.16-2.27		2.64	2.48-2.82
2007	2.23	2.18-2.29		2.16	2.11-2.22		2.61	2.46-2.76
2008	2.28	2.23-2.33		2.21	2.16-2.26		2.66	2.52-2.82
*P*	< 0.001			< 0.001			0.084	

**1:**
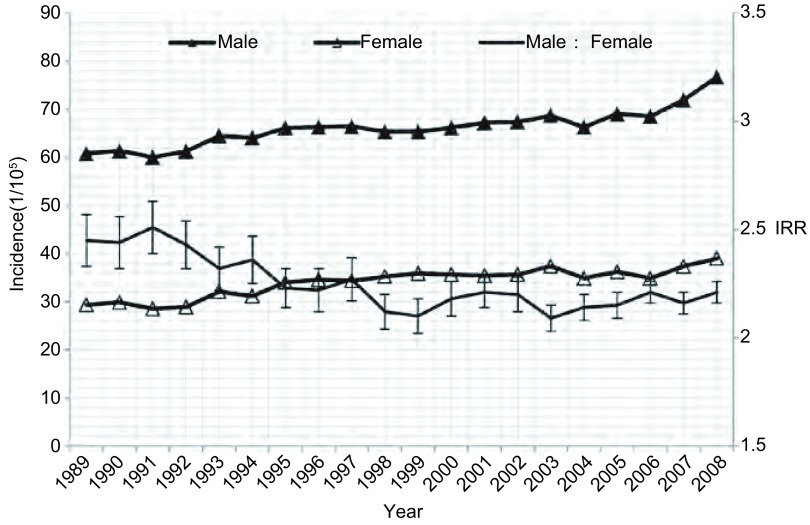
1989年-2008年中国城市地区肺癌发病率及男女发病比变化 The trend of lung cancer incidence rate ratio (IRR) between male and female in urban in China, 1989-2008

**2:**
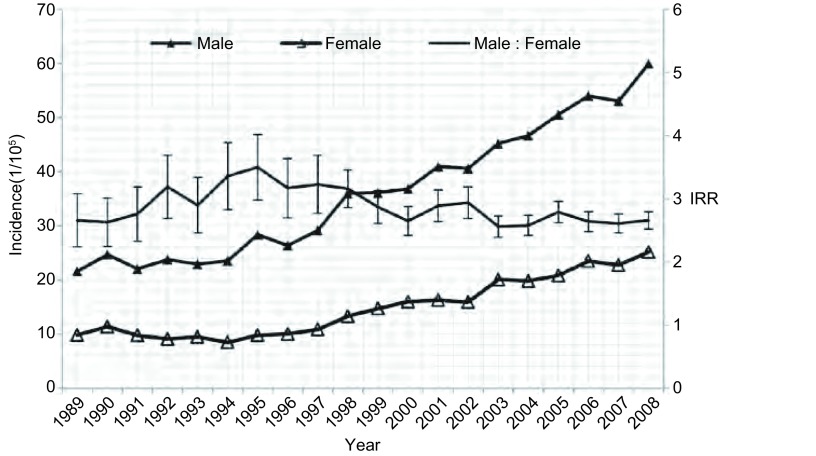
1989年-2008年中国农村市地区肺癌发病率及男女发病比变化 The trend of lung cancer IRR between male and female in rural in China, 1989-2008

### 2.2 肺癌发病率城乡比变化

1989年-2008年期间，我国城市地区的肺癌年龄调整发病率均高于农村地区，且城市男性和女性的发病率也均明显高于农村地区的男性和女性。但在此期间，肺癌发病率的城乡比从1989年的2.07下降到2008年的1.14，呈明显下降趋势（*P* < 0.001）；其中男性肺癌发病率的城乡比从1989年的1.99下降到2008年的1.07，女性肺癌发病率的城乡比从1989年的2.25下降到2008年的1.32，男性和女性的发病率城乡比均呈明显下降趋势（*P* < 0.001），城乡差异明显缩小（表 2，图 3和图 4）。

**2:** 1989年-2008年中国肺癌发病率城乡比值 Incidence rate ratio (IRR) of lung cancer between urban and rural areas in China, 1989-2008

Year	Total			Male			Female	
	IRR	95%CI		IRR	95%CI		IRR	95%CI
1989	2.07	1.91-2.25		1.99	1.80-2.19		2.25	1.94-2.60
1990	1.79	1.66-1.93		1.72	1.57-1.89		1.93	1.68-2.21
1991	1.97	1.82-2.13		1.89	1.71-2.07		2.15	1.86-2.48
1992	1.84	1.70-1.99		1.67	1.53-1.83		2.27	1.96-2.64
1993	2.08	1.92-2.25		1.90	1.73-2.08		2.52	2.18-2.91
1994	2.08	1.92-2.25		1.84	1.68-2.02		2.76	2.36-3.21
1995	1.79	1.67-1.92		1.54	1.41-1.67		2.54	2.20-2.93
1996	1.80	1.67-1.94		1.58	1.45-1.73		2.39	2.08-2.76
1997	1.64	1.53-1.76		1.44	1.33-1.57		2.20	1.92-2.52
1998	1.56	1.49-1.63		1.36	1.29-1.44		2.11	1.93-2.30
1999	1.53	1.46-1.60		1.36	1.29-1.44		1.94	1.78-2.11
2000	1.60	1.53-1.67		1.48	1.40-1.56		1.88	1.74-2.04
2001	1.46	1.40-1.52		1.32	1.25-1.39		1.81	1.67-1.96
2002	1.46	1.40-1.52		1.31	1.25-1.38		1.84	1.70-1.99
2003	1.33	1.29-1.38		1.23	1.18-1.29		1.55	1.46-1.65
2004	1.38	1.33-1.42		1.28	1.24-1.34		1.59	1.50-1.69
2005	1.23	1.19-1.27		1.13	1.09-1.17		1.48	1.40-1.57
2006	1.11	1.08-1.15		1.05	1.01-1.09		1.27	1.20-1.34
2007	1.17	1.14-1.21		1.10	1.06-1.14		1.35	1.28-1.42
2008	1.14	1.11-1.17		1.07	1.03-1.10		1.32	1.26-1.39
*P*	< 0.001			< 0.001			< 0.001	

**3:**
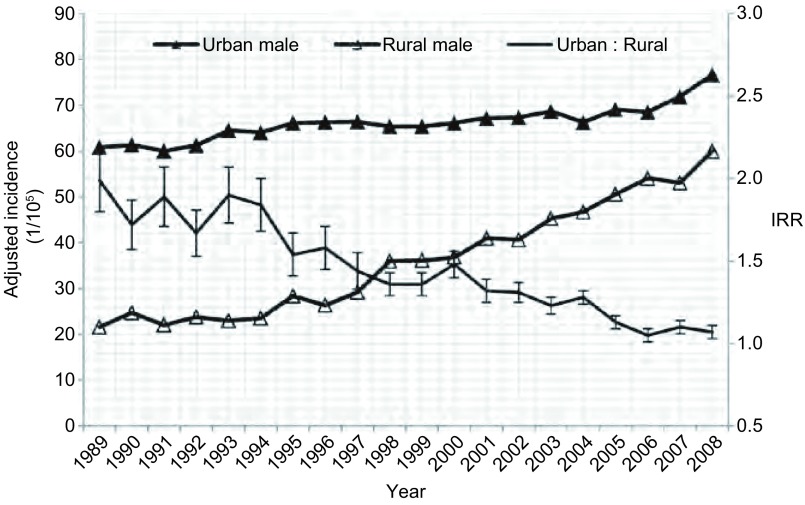
1989年-2008年中国男性城乡肺癌发病率及城乡发病比变化 The trend of lung cancer IRR between urban and rural areas in male in China, 1989-2008

**4:**
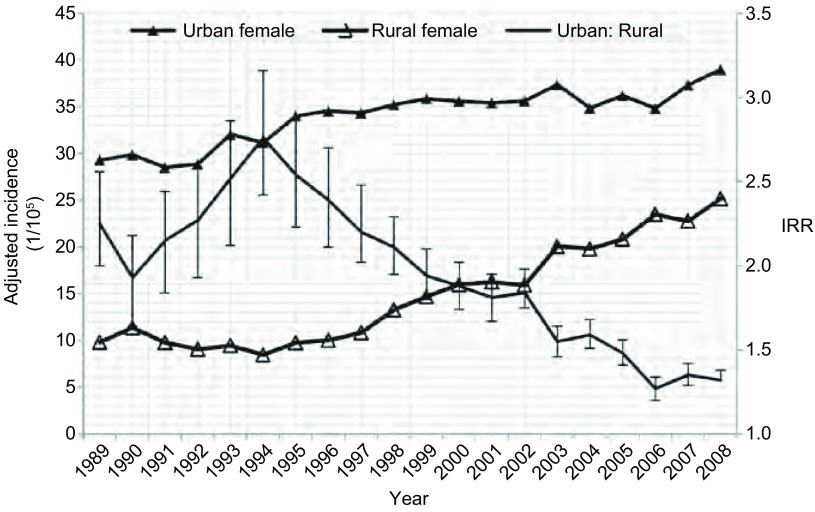
1989年-2008年中国女性肺癌城乡发病率及城乡发病比变化 The trend of lung cancer IRR between urban and rural areas in female in China, 1989-2008

### 2.3 肺癌平均发病年龄变化

1989年，全国肿瘤登记地区男性和女性肺癌的平均发病年龄分别为65.32岁和65.14岁，其中城市男性和女性的平均发病年龄分别为65.51岁和65.34岁，农村男性和女性分别为63.07岁和62.63岁。到了2008年，登记地区男性和女性的肺癌发病平均年龄分别为67.87岁和68.05岁，其中城市男性和女性分别为68.19岁和68.34岁，农村男性和女性分别为66.40岁和66.34岁。全国男性和女性肺癌平均发病年龄较1989年分别升高了2.55岁和2.91岁，城市男性和女性分别升高了2.68岁和3.00岁，农村男性和女性分别升高了3.33岁和3.71岁。20年间，肺癌的平均发病年龄无论在城市和农村、男性和女性中均随着时间推移呈明显升高趋势（*P* < 0.001）（表 3、图 5）。

**3:** 1989年-2008年中国肺癌平均发病年龄 Average age of lung cancer incidence in China, 1989-2008

Year	All areas			Urban			Rural	
	Male	Female		Male	Female		Male	Female
1989	65.32	65.14		65.51	65.34		63.07	62.63
1990	65.53	65.33		65.74	65.49		63.31	63.57
1991	66.02	65.32		66.22	65.41		63.79	64.28
1992	66.13	66.06		66.36	66.20		63.60	64.13
1993	66.30	66.09		66.52	66.37		63.77	62.19
1994	66.31	66.09		66.51	66.36		64.12	61.98
1995	66.76	66.44		67.06	66.76		63.90	61.78
1996	66.92	66.66		67.22	66.93		63.84	62.82
1997	67.19	67.14		67.54	67.42		63.90	63.55
1998	66.49	66.30		66.92	66.66		64.46	63.86
1999	66.78	66.98		67.26	67.45		64.53	64.06
2000	67.20	67.34		67.61	67.81		64.87	63.99
2001	67.11	67.48		67.57	67.94		64.63	64.31
2002	67.23	67.47		67.65	67.91		64.98	64.24
2003	67.12	67.11		67.36	67.44		65.87	65.07
2004	67.20	67.06		67.51	67.38		65.67	65.17
2005	67.30	67.11		67.48	67.30		66.50	66.08
2006	67.33	67.44		67.47	67.74		66.58	65.55
2007	67.63	67.82		67.81	68.16		66.84	66.07
2008	67.87	68.05		68.19	68.34		66.40	66.34
*P*	< 0.001	< 0.001		< 0.001	< 0.001		< 0.001	< 0.001

**5:**
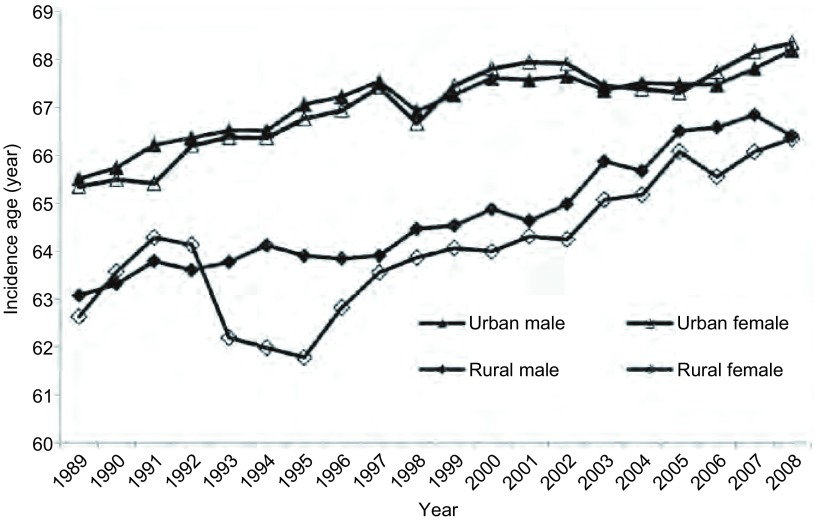
1989年-2008年中国肺癌平均发病年龄变化 Trend of average age of lung cancer incidence in China, 1989-2008

## 3 讨论

肺癌是危害我国公众健康的主要恶性肿瘤之一。随着我国社会经济的飞速发展，居民生活方式的急剧转变，人口老龄化进程的不断加快，工业化和城市化进程的快速推进所带来的空气污染不断加剧，以及人群吸烟率的居高不下，我国肺癌的危害呈不断加剧趋势。21世纪初全国第三次死因回顾抽样调查显示，我国2004年-2005年肺癌的死亡率为30.84/10万，较20世纪70年代第一次死因调查的5.60/10万和90年代第二次死因调查的15.19/10万均明显升高，在癌症死亡中的构成也由7.35%和16.20%提高至22.70%，癌症死亡顺位也由第5位和第3位跃居至第1位^[5]^。因此，面对我国肺癌负担不断加重的严峻形势，应将肺癌防治研究工作列为恶性肿瘤防控的重点。为了制定有效的肺癌防治策略，需动态掌握我国近几十年来不同地区，不同人群肺癌的流行规律及其变化趋势，以有针对性地开展防治工作。

一直以来，我国城市和农村地区在经济发展水平、医疗保健资源、生活方式改变、工业化带来的空气污染等各方面均存在较大差异，肺癌发病水平及其在癌症谱中的地位也不尽相同。以往的研究发现，我国城市地区肺癌流行趋势表现为发达国家的特征，其发病水平高于其他癌种，较农村地区也明显较高，而农村地区仍呈现发展中国家特征，以上消化道癌最为高发，而肺癌发病相对较低^[3]^。本文对1989年-2008年我国肺癌发病率城乡差异的变化趋势分析发现，尽管我国城市地区的肺癌发病水平仍高于农村地区，但肺癌发病率的城乡差异随时间的推移呈明显下降趋势，城乡发病率比从1989年的2.07下降到2008年的1.14，男性和女性的肺癌城乡发病率比也分别从1989年的1.99和2.25下降到2008年的1.07和1.32，城乡差异明显缩小，甚至接近。这可能是随着我国农村地区经济的发展，城镇化步伐的不断加快，工业化不断在向农村地区的蔓延，越来越多农村居民的生活方式和居住环境有不断向城市化转变的趋势；与此同时，农村与城市地区相比吸烟率更高^[6]^，均导致了农村地区的肺癌发病率呈现快速升高趋势，其增幅大于城市地区所致。之前有学者^[7]^对美国1950年-2007年肺癌死亡率的城乡变化趋势进行过研究，发现随着城市吸烟率较农村的快速下降，城市和农村肺癌的死亡率高低差异呈现逆转态势，目前美国农村地区的肺癌死亡率明显高于城市地区。

吸烟是肺癌的最主要危险因素。研究表明，全球80%-90%的肺癌死亡可归因于吸烟。2010年全球成人烟草调查^[6]^显示，与1996年和2002年相比，我国男性的吸烟率仍居高不下，这可能是我国男性肺癌发病率多年来不断升高的重要原因。与此同时，该调查显示我国女性的吸烟率没有明显改变，仍维持很低的水平。因此，单从吸烟率角度就不能很好解释本次研究发现的，我国女性肺癌发病率近20年来较男性升幅更大，肺癌发病男女性别比呈明显下降趋势的现状。这可能是不同性别的生理或遗传差别所致。有研究^[8, 9]^表明，雌激素的影响，对烟草中致癌物的敏感性较男性更高，环境烟草烟雾暴露，病毒感染等是导致女性在低吸烟率情况下肺癌发病率持续升高的重要因素，但还需进一步的病因学研究加以证实。

本次研究还发现，1989年-2008年我国肺癌的平均发病年龄呈明显增高趋势，全国男性提高了2.55岁，女性提高了2.91岁，特别是农村女性，由1989年的62.63岁升增长到2008年的66.34岁。这与我国目前的人口老龄化，期望寿命增加有关。有研究^[10]^表明，老龄化是恶性肿瘤最重要的独立危险因素，因为在某种程度上，人的生存年龄越长，暴露于致癌物和受到基因损伤的持续时间就越长，恶性肿瘤发生的概率就越大，这对肺癌也不例外。国内学者对我国1991年-1998年肺癌死亡率升高的原因进行分析发现，除外其他危险因素水平变化的影响，近50%的肺癌死亡率升高可归因于老龄化^[11]^。因此，我国人口老龄化进程加快带来的肺癌负担不断加重的问题不容忽视。

近几年来，我国肿瘤登记工作快速推进，登记覆盖地区不断扩大，数据质量也稳步提高。为卫生行政部门肿瘤防治重点的决策，科研部门肿瘤病因学的相关研究提供了科学的人群基础数据。本文的研究发现正是得益于此。针对目前我国肺癌发病的城乡差异和性别差异已明显缩小，肺癌平均发病年龄明显推后的现状，今后的防治重点应有所调整。应兼顾城乡人群烟草流行的不同特点，有针对性地开展形式多样的控烟工作，降低人群吸烟率，进而减少女性在环境烟草烟雾暴露的机会，以控制甚至降低人群肺癌发病不断升高的趋势。与此同时，应加强对65岁以上肺癌多发年龄人群的关注，着力研究有效的肺癌早期筛查技术，争取将肺癌防治的关口前移，降低肺癌随着我国老龄化步伐不断加快产生的巨大负担。
